# Exploring the Prevalence of Post-traumatic Stress Disorder and Post-traumatic Stress Symptoms in Parents Within 12 Months of Child Burn Injury: A Systematic Review

**DOI:** 10.1093/jbcr/irae033

**Published:** 2024-02-29

**Authors:** Naomi Hope Chouinard, Davaine Joel Ndongo Sonfack, Sue-Ling Chang, Frédéric Bergeron, Chanel Beaudoin Cloutier, Jason Robert Guertin

**Affiliations:** Département de médecine sociale et préventive, Faculté de médecine, Université Laval, Québec, G1V 5C3, Canada; Centre de recherche du CHU de Québec-Université Laval, Québec, G1S 4L8, Canada; Centre de recherche en organogénèse expérimentale de l’Université Laval/LOEX, Québec, G1J 5B3, Canada; Centre de recherche du CHU de Québec-Université Laval, Québec, G1S 4L8, Canada; Centre de recherche en organogénèse expérimentale de l’Université Laval/LOEX, Québec, G1J 5B3, Canada; Département de chirurgie, Faculté de médecine, Université Laval, Québec, G1V 5C3, Canada; Département de médecine sociale et préventive, Faculté de médecine, Université Laval, Québec, G1V 5C3, Canada; CHU de Québec-Université Laval, Québec, G1S 4L8, Canada; Bibliothèque-Direction des services-conseils, Université Laval, Québec, G1V 5C3, Canada; Centre de recherche du CHU de Québec-Université Laval, Québec, G1S 4L8, Canada; Centre de recherche en organogénèse expérimentale de l’Université Laval/LOEX, Québec, G1J 5B3, Canada; Département de chirurgie, Faculté de médecine, Université Laval, Québec, G1V 5C3, Canada; Département de médecine sociale et préventive, Faculté de médecine, Université Laval, Québec, G1V 5C3, Canada; Centre de recherche du CHU de Québec-Université Laval, Québec, G1S 4L8, Canada; Centre de recherche en organogénèse expérimentale de l’Université Laval/LOEX, Québec, G1J 5B3, Canada

**Keywords:** burns, parent, pediatric burn patients, mental health, post-traumatic, caregiver, trauma

## Abstract

Our systematic review aimed to investigate the prevalence of post-traumatic stress symptoms (PTSS) and post-traumatic stress disorder (PTSD) among parents within 12 months of their child’s burn injury. A literature search was conducted in PubMed, Embase, Web of Science, Psychinfo, and CINAHL on January 6, 2023, for quantitative studies reporting the prevalence of PTSD and/or PTSS in parents within 12 months following their child’s burn injury. The risk of bias was assessed using the Mixed Methods Appraisal Tool version 2018. A narrative synthesis of prevalence was presented. We identified 15 articles that met our inclusion criteria. The prevalence of PTSS within 12 months following the burn injury ranged from 6% to 49%. Prevalence estimates of PTSD within the 12 months following a burn injury were limited, ranging from 4.4% to 22%. Our findings highlight the significant impact of burn injuries on parental mental health, with a considerable proportion of parents experiencing PTSS within 12 months following their child’s burn injury. Prevalence estimates for PTSD were limited and warrant further investigation. Our review also underscores the need for standardization of PTSS/PTSD terminology. Timely and targeted psychological support is needed for parents in the aftermath of their child’s burn injury.

## INTRODUCTION

Burn injuries are irrevocably among the most burdensome to patients and health systems. The experience of sustaining burn injuries can be an incredibly distressing event. The psychological impact of such traumatic events on burn survivors, both adults and children, is an area of increasing interest.^1–3^ Specifically, post-traumatic stress disorder (PTSD) has gained recognition as an important factor that warrants closer examination. PTSD is a mental health problem that is diagnosed at least a month following a terrible event (eg, a child’s burn injury).^4^ In some cases, patients’ PTSD will be preceded by an acute stress disorder (ASD) (a diagnosis occurring within 3 days to 1 month of the event), which will evolve into PTSD.^5^

Fortunately, burn injuries are relatively rare in children and seem to be decreasing in certain countries.^6^ Burn mortality has also decreased significantly, attributable to notable advances in the field, such as regionalization of burn care, improved understanding of burn pathophysiology, and various aspects of burn treatment such as burn assessment, transplantation, infection control, and nutritional support.^7^ This improved survival, therefore, implies new challenges that go far beyond those mobilized during the acute phase of treatment. Severe burns can be considered a chronic disease with many secondary comorbidities besides the care to treat the burns per se, such as increased risks of anxiety and depression.^8^

While several studies have focused on the psychosocial impacts of burns in patients,^9–11^ few studies have looked at distress among family caregivers.^12,13^ In particular, the psychological distress of parents of children with burns is poorly quantified in the literature. According to a scoping review, family members of burn survivors generally have concerns about the new roles they must fulfill, as well as financial and psychological concerns.^14^ Parents of children with burns live with guilt, blame, and shame.^14^

More importantly, a few studies suggest that a significant proportion of parents may develop PTSD or post-traumatic stress symptoms following their child’s burn injury (ie, ^15,16^). Symptoms of PTSD are often grouped into 4 broad families: intrusive memories, avoidance behaviors, negative changes in thoughts and moods, and changes in physical and emotional responses.^4^ Post-traumatic stress symptoms (PTSS), while not a formal diagnosis, evaluate the presence of main PTSD symptoms (ie, those listed above).^17^ Therefore, PTSS serves as a proxy for PTSD. Questionnaires evaluating PTSS generally use a predetermined cutoff for clinically significant symptoms, but they do not represent a diagnostic tool for PTSD.^18^ McGarry et al. showed that parents of children with burns suffered significantly more PTSS than the general population in the months following the injury.^15^ Another study found that nearly half of the parents of children with severe burns had significant PTSS within 3 months of the injury.^16^ This usually involved parent–child conflict, parental dissociation, and PTSS in their child who sustained a burn injury.^16^

Despite the magnitude of these numbers, to our knowledge, no systematic review has ever been conducted on PTSD or PTSS in this population. A better understanding of the prevalence of PTSD and PTSS in parents of children with burns could raise awareness of the importance of this health problem that extends beyond the patient. While care for the burn patient is often intensive in the acute phase following injury, distress may persist in parents in the months following the burn.^16^

### Objective

The objective of this systematic review is to assess the psychological distress of parents in terms of PTSD and PTSS within 12 months following a burn injury to their child.

## METHODS

The protocol of this systematic review was published on PROSPERO (ID CRD42023413319). This systematic review is in accordance with the *Preferred reporting items for systematic reviews and meta-analysis statement* (PRISMA) guidelines.^19,20^ The PRISMA checklist, which serves as the basis for the format of this review, can be found in Supplementary Appendix A.

### Search strategy and inclusion criteria

The studies included in this review met the following criteria: (1) studies assessing the prevalence of PTSD and/or PTSS in parents whose child (18 years and under) has suffered a burn over a period of 12 months or less following the child’s burn injury; (2) quantitative observational study designs. Our review had no exclusion criteria.

A literature search was performed without restrictions on January 6, 2023, by searching the following bibliographic databases: PubMed, Embase (Embase.com), Web of Science, PsycInfo (Ovid), and CINAHL. An additional search was also conducted on Google Scholar search engine. Known websites have been consulted to find grey literature, and references of included articles were also consulted. Since the topic did not lend itself to a clinical trial context, registries were not considered for the purposes of this review.

The search strategy was built around concepts of burns, PTSD/PTSS, and parents. A mix of words from free and controlled vocabulary was used for each of our 3 concepts. Our research strategy retrieved 708 citations. Details of the search strategy for each database can be found in Supplementary Appendix B.

### Selection process and data extraction

All references were exported to the *Covidence* software (Melbourne, Australia) for the selection process, where duplicates were removed using the software’s automatic function.

To ensure a clear understanding of the selection criteria based on titles and abstracts, a pilot phase with a subgroup of 50 articles was initiated first with 2 reviewers (NHC and DJNS). The process was reproduced until a kappa value of 0.7 was obtained, which indicated a good understanding and a high level of agreement among the chosen studies. The screening of articles was done by 2 individuals independently. Each reviewer determined if the criteria as previously defined were met when selecting based on titles and abstracts. If this was the case for both reviewers, then the article proceeded to the next step, that is, the selection based on the full text. If there was a discrepancy between the 2 reviewers, a third reviewer (SLC), would resolve conflicts. The same process regarding conflicts between reviewers was adopted for full-text review.

### Data collection process

For data collection, a codification guide was available to both reviewers. This process began with a pilot phase where 2 independent reviewers extracted data from a subgroup of articles. Following this pilot phase, data collection was extended to all articles. A third reviewer resolved discrepancies. Study authors were contacted on 3 separate occasions to request missing information.

### Outcomes

The outcomes were divided into 2 categories: the prevalence of PTSD and the prevalence of PTSS. Data extracted included the questionnaire used to assess both PTSS and/or PTSD prevalence, the method of assessment, and the cutoff for the clinically significant PTSS or PTSD according to the questionnaire. Moreover, the prevalence was documented for up to 5 time points. All prevalence points were measured only for the parent(s) who completed the questionnaire (self-reported).

Key study characteristics were extracted: first author, publication year, and study design. Population characteristics data were also collected, including sample size, number of fathers (as defined by the manuscript), country of study, mean TBSA of children’s burns, and mean age of children with burns. All missing data were reported as such.

### Risk of bias assessment

Risk of bias assessment was conducted using the Mixed Methods Appraisal Tool (MMAT) version 2018. This tool can be used to assess the risk of bias in systematic reviews of quantitative, qualitative, and mixed methods studies. As all our studies were quantitative, section 4 of the MMAT was used.^21^ Criteria in this section examined the sampling strategy, sample representativeness, appropriate measures, risk of nonresponse bias, and appropriate statistical analysis. Moreover, this appraisal tool assessed the research question and the appropriateness of collected data to answer the research question. A global assessment based on the criteria was used instead of a total risk score, as the tool does not provide specific cutoffs for risk of bias level.^21^ The reviewers evaluated the risk of bias independently and determined if the risk of bias for selected articles was high (≥4 no answers), moderate (2-3 no answers), low (1 no answer or only yes answers), or unclear (>0 cannot tell answers).

### Synthesis methods

A narrative synthesis was undertaken since heterogeneity was too high between studies for a meta-analysis. Prevalence was categorized according to measure (PTSS or PTSD) and according to the time it was assessed. When available, prevalence was reported for both mothers and fathers separately. The risk of bias assessment was also considered in our result interpretation. Our results were reanalyzed by excluding these studies to ensure the reliability and accuracy of our findings.

In this review, only factual data as presented by the authors of the included studies was reported. In order to present findings transparently and objectively, no data interpretation was conducted, ensuring that the information reported accurately reflects the original data provided by the authors.

## RESULTS

**Figure 1.**
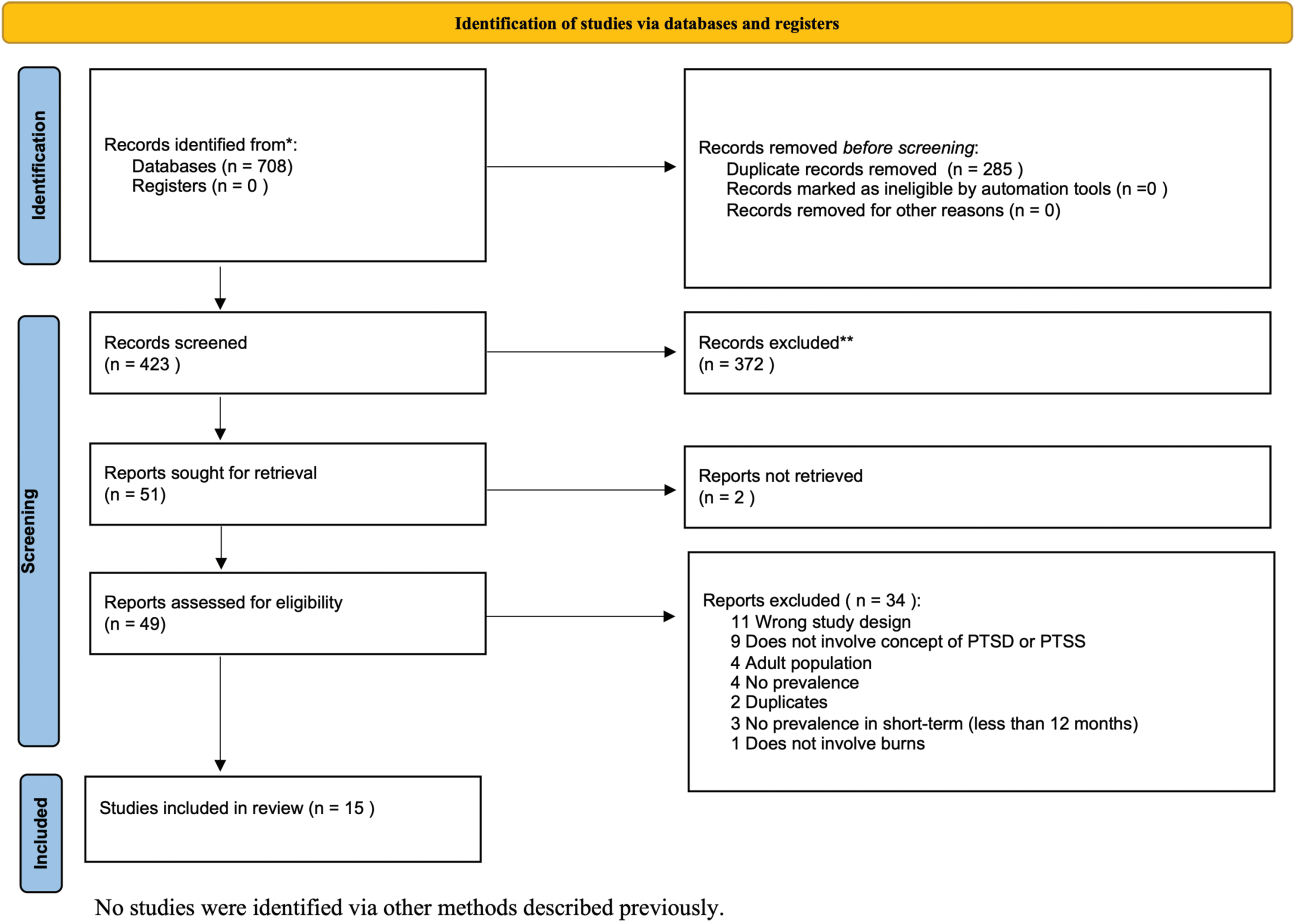
PRISMA Flow Chart. Preferred Reporting Items for Systematic Reviews and Meta-analysis Statement (PRISMA) Flow Chart of Included Articles

Our database research strategy yielded a total of 708 studies, of which 285 duplicates were removed (see Figure 1). Our review included 15 articles that met our inclusion criteria. Despite screening the references of selected studies and searching through grey literature, no additional studies were identified. Of note, we set an age limit of 18 years for the children with burns included in our analysis, which led to exclusions of articles including ages higher than 18. Consequently, our review did not include a recent study investigating PTSS in parents with a child who sustained a burn injury up to 19.5 years old.^22^

Characteristics of the included studies are presented in Table 1. Among the 15 included articles, 9 evaluated PTSS prevalence,^16–18,24–29^ 5 evaluated PTSD prevalence,^15,30–33^ and 1 evaluated both.^23^ The studies included in our review were mostly conducted in the United States^16,23,32,33^ and Europe.^17,18,24,25,28,29^ Respondent sample size within included studies ranged varied from 16 to 407. Mean %TBSA in children ranged from a low of 1.85% to a high of 16.93%. The age range of children with burns in our included studies was 0-18 years old, with a particular focus on children under 6 years old in 6 of the 15 studies.^18,23,26,27,31,33^

**Table 1.** Characteristics of Included Studies, According to the Measure of Prevalence of Post-traumatic Stress Symptoms (PTSS) or Post-traumatic Stress Disorder (PTSD)

First author and publication year	Name of country	Sample size	Number of fathers	Mean %TBSA of children burns	Age range of children with burns	Questionnaire for PTSS or PTSS measurement
**Post** **-** **traumatic** **stress** **symptoms**						
Hall (2006)^a^^,16^	United States	62	8	16.93	6-17 y	PCL-C
Bakker (2010)^17^	The Netherlands	48	0	NA	0-13 y	IES validated Dutch version
Bakker (2013)^18^	The Netherlands; Belgium	345	159	7.5	0.7-4.6 y	IES validated Dutch version
Odar (2013)^23^	United States	45	8	2.67	0.23-4.6 y	PCL-S
Egberts (2016)^24^	The Netherlands; Belgium	155	65	10.0	9.5-17.8 y	IES validated Dutch version
Egberts (2017)^25^	The Netherlands; Belgium	202	108	9.6	7.9-17.8 y	IES validated Dutch version
Brown (2019)^26^	Australia	87	14	1.87	1-6 y	PC-PTSD
Brown (2019)^27^	Australia	83	14	1.85	1-6 y	PC-PTSD
Hawkins (2019)^b^^,28^	United Kingdom	91	25^c^	3.88	0.33-15 y	IES-R
Egberts (2020)^29^	The Netherlands; Belgium	296	0	8.17	0.67-4 and 8–18 y	IES validated Dutch version
**Post** **-** **traumatic stress** **disorder**						
Fukunishi (1998)^30^	Japan	16	0	NA	NA	Structured clinical interview for DSM-111-R
McGarry (2013)^^d^,15^	Australia	63	17	4.12	0.67-16 y	IES-R and IES
Odar (2013)^e^^,23^	United States	45	8	2.67	0.23-4.6 y	PCL-S
De Young (2014)^31^	Australia	120	9	3.16	1-6 y	PDS; version Foa 1995
Parrish (2019)^32^	United States	407	NA^f^	4.15	NA	SPRINT
Tully (2022)^d^^,31^	United States	57	NA^g^	2.4	0-5 y	PCL-5

Abbreviations: IES, the impact of event scale; IES-R, the impact of events scale-revised; PCL-5, posttraumatic checklist—5th edition; PCL-C, PTSD checklist; PCL-S, PTSD checklist stressor-specific version; PC-PTSD, the primary care-post-traumatic stress disorder screen; PDS, posttraumatic diagnostic scale; SPRINT, short posttraumatic stress disorder rating interview.

^a^The article used PTSS, PTSD, and PTSD interchangeably while only presenting one prevalence measure. Title included PTSS and was categorized as such.

^b^PTSS was defined as post-traumatic stress syndrome rather than PTSS.

^c^The sample included 63 mothers, 25 fathers, and 3 other primary caregivers.

^d^The article evaluated PTSD symptoms but interpreted prevalence as PTSD.

^e^The article evaluated the prevalence of PTSS and PTSD and is, therefore, in both categories.

^f^The article mentioned prevalence measured in the short term, but no specification was given.

^g^Authors report 81.4% mothers, 15.3% fathers, and 3.4% other caregivers.

For measurement of PTSS, 5 studies used “The Impact of Event Scale” (IES); validated Dutch version^17,18,24,25,29^ and one used the revised version (IES-R).^28^ One study used the “PTSD Checklist” (PCL-C),^16^ while another study used the “PTSD Checklist Stressor-Specific version” (PCL-S).^23^ Two studies used “The Primary Care-Post-Traumatic Stress Disorder” (PC-PTSD).^26,27^ For PTSD assessment, all studies used their own validated questionnaires. An article used both the Impact of Events Scale and the Revised Version.^15^

A narrative analysis of PTSS prevalence can be found in Table 2 (a graphical representation can be found in Supplementary Appendix C). Some articles were not included in the quantitative data synthesis as they referred to the same cohort (Bakker 2013^18^; Egberts 2016^24^; Brown 2019^27^). Articles with distinct prevalence for women and men and larger samples were prioritized among repeated cohorts. However, 2 articles with overlapping cohorts were included, as one compared the prevalence in mothers vs fathers (Egberts 2017^25^), and the other had a larger sample (Egberts 2020^29^). The 12-month prevalence of PTSS ranged from 6% to 49%, with a higher concentration of studies observing PTSS within the first 3 months following a burn injury. Results also suggested that PTSS among parents decreased over time. Mothers exhibited a higher prevalence of PTSS compared to fathers (ie, 48% vs 26% within 1 month after burn injury^25^). However, one study estimated a higher prevalence of PTSS in men as opposed to women (40% vs 32.8%^28^).

**Table 2.** Post-traumatic Stress Symptoms (PTSS) Prevalence in Parents Within 12 Mo Following a Burn Injury to Their Child

First author and publication year^a^	Sample size	Time of measurement after injury	Prevalence (%)
Hall (2006)^16^	62	3 mo	47
Bakker (2010)^17^	48	12 mo	42
Odar (2013)^23^	45	0.5-12 mo. Average: 72.53 days	29
Egberts (2017)^25^			
Mothers	108	≤1 mo^b^	48
	89	3 mo	29
	68	12 mo	25
Fathers	84	≤1 mo^b^	26
	73	3 mo	14
	48	12 mo	6
Brown (2019a)^26^	8	First dressing change (average 3.24 days)	11
Hawkins (2019)^28^			
Women	NA^c^	≤2 mo^b^	32.8
Men	NA^c^	≤2 mo^b^	40
Egberts (2020)^d^^,29^	296	≤1 mo^b^	49

^a^Some articles were not included in the quantitative data as they referred to the same cohort: Bakker 2013^18^; Egberts 2016^24^; Brown 2019.^27^

^b^≤ Implies that prevalence was not measured at a specific time point but rather in a time window.

^c^The sample included 63 mothers, 25 fathers, and 3 other primary caregivers.

^d^This study overlaps some participants of Egberts 2017.

Similarly, PTSD prevalence in parents within 12 months following a burn injury to their child is reported in Table 3 (graphical representation can be found in Supplementary Appendix D). The prevalence of PTSD varied between 4.4% and 22%. Of note, studies included in this analysis did not extend beyond 6 months, and none of them reported PTSD prevalence specifically for mothers vs fathers or women vs men. Furthermore, our data for PTSD did not reveal any clear trend in terms of changes in prevalence over time. There was less available evidence regarding the prevalence of PTSD compared to the prevalence of PTSS.

**Table 3.** Post-traumatic Stress Disorder (PTSD) Prevalence in Parents Within 12 Months Following a Burn Injury to Their Child

First author and publication year	Sample size	Time of measurement after injury	Prevalence (%)
Fukunishi (1998)^30^	16	≤0.5 mo^a^^,^^b^	18.8
McGarry (2013)^15^	63	3 mo	21.3
Odar (2013)^23^	45	0.5-12 mo^b^ Average: 72.53 days	4.4
De Young (2014)^31^	120	1 mo	22
	120	6 mo	5
Parrish (2019)^32^	407	*Unclear* ^c^	12.53
Tully (2022)^33^	57	3 mo	10.5

^a^≤ Implies that prevalence was not measured at a specific time point but rather in a time window.

^b^As PTSD can only be diagnosed 1 month after trauma, any PTSD diagnosis before 1 month would probably more accurately represent an acute stress disorder (ASD) diagnosis.

^c^The article mentioned prevalence measured in the short term, but no specification was given.

The risk of bias assessment according can be found in Table 4. The risk of bias was generally low,^15,17,18,24–27,29–31^ with a few articles having a moderate risk^16,23,28,33^ of bias with only one article identified as having a high risk of bias.^32^ Generally, a relevant sampling strategy and the representativeness of the sample regarding the target population were more problematic. One study had a high risk of bias and unclear assessment time.^32^ This study’s PTSD prevalence did not affect the range of prevalence observed in our study. As we did not conduct a meta-analysis, there was no need to reanalyze the data.

**Table 4.** Risk of Bias Assessment (Mixed Methods Appraisal Tool version 2018^a^)

First author and publication year	Are there clear research questions?	Do the collected data allow to address the research questions?	Is the sampling strategy relevant to address the research question?	Is the sample representative of the target population?	Are the measurements appropriate?	Is the risk of nonresponse bias low?	Is the statistical analysis appropriate to answer the research question?	Assess risk of bias based on previous questions
**Post** **-** **traumatic** **stress** **symptoms**								
Hall (2006)^16^	No	Yes	No	No	Yes	Yes	Yes	Moderate
Bakker (2010)^17^	Yes	Yes	No	Yes	Yes	Yes	Yes	Low
Bakker (2013)^18^	Yes	Yes	Yes	Yes	Yes	Yes	Yes	Low
Odar (2013)^23^	Yes	Yes	No	No	No	Yes	Yes	Moderate
Egberts (2016)^24^	Yes	Yes	Yes	Yes	Yes	Yes	Yes	Low
Egberts (2017)^25^	Yes	Yes	Yes	Yes	Yes	Yes	Yes	Low
Brown (2019)^26^	Yes	Yes	Yes	No	Yes	Yes	Yes	Low
Brown (2019)^27^	Yes	Yes	Yes	No	Yes	Yes	Yes	Low
Hawkins (2019)^28^	Yes	Yes	Yes	No	No	Yes	Yes	Moderate
Egberts (2020)^29^	Yes	Yes	Yes	Yes	Yes	Yes	Yes	Low
**Post** **-** **traumatic** **stress** **disorder**								
Fukunishi (1998)^30^	Yes	Yes	Yes	Yes	Yes	Yes	Yes	Low
McGarry (2013)^15^	Yes	Yes	No	Yes	Yes	Yes	Yes	Low
Odar (2013)^23^	Yes	Yes	No	No	No	Yes	Yes	Moderate
De Young (2014)^31^	Yes	Yes	No	Yes	Yes	Yes	Yes	Low
Parrish (2019)^32^	Yes	No	No	Yes	No	No	Yes	High
Tully (2022)^33^	Yes	Yes	Yes	No	No	Yes	Yes	Moderate

^a^Quan Nha et al.^21^

## DISCUSSION

Our systematic review aimed to examine the prevalence of PTSS and PTSD among parents within the 12 months following a burn injury to their child. A total of 15 articles met our inclusion criteria.^15–18,23–33^ Prevalence of PTSS within 12 months of the burn injury ranged from 6% to 49%, and a downward trend was observed with time since the burn injury (Table 2). PTSD prevalence within the 12 months following a burn injury was limited, with numbers ranging from 4.4% to 22% (Table 3). There were no clear patterns or trends in terms of changes in the prevalence of PTSD over time, and no study examined the prevalence of PTSD in mothers vs fathers.

Providing psychological support for parents during this time appears to be a crucial step to help the child’s recovery as well as their own. Indeed, research has shown that psychological distress experienced by parents poses a significant challenge as it can also affect the recovery of the child who sustained a trauma. For example, a study has shown that parental PTSS 6 weeks following child trauma was a strong predictor of child PTSS.^34^ Similar trends have also been observed in parents of children who sustained a burn injury. More specifically, Haag et al. have shown that maternal acute stress following a child’s burn injury has a significant impact on the child’s psychological reaction.^35^ Additionally, Stoddard et al. found that parent’s acute stress symptoms are an important risk factor for acute stress symptoms in children with burns.^36^

Data from our studies was mostly regarding smaller burns, that is, low %TBSA, which is a criterion of burn severity (Table 1). However, a recent article analyzing the World Health Organization Global Burn Registry found that among the available data from 20 countries, 52% of pediatric burns were of at least %TBSA ≥15.^37^ Given that the average %TBSA in the studies included in our review was notably low, it is plausible to hypothesize that the prevalence of PTSS and PTSD among parents of children who experience burn injuries is underestimated. Although the rationale for excluding severe cases was not always explained, it may partially be due to ethical concerns (ie, approaching parents for such studies after the child’s death or when the child is in intensive care). As such, many articles focused on outpatients or less severe cases. The nonrepresentativeness of samples in terms of burn severity also increased the risk of bias in our studies, as it was a criterion considered in our appraisal tool.

The World Health Organization Global Burn Registry data also indicated that the average age of burned pediatric patients was 5.3 years old, whereas older children had more severe injuries.^37^ Six out of fifteen included studies concentrated on children under the age of 6, which is consistent with the previously described average age (5.3 years old). Our studies, therefore, accurately represent the typical age range of burn patients in the pediatric population.

Furthermore, in the articles evaluating PTSS prevalence, some studies examined how PTSS prevalence differed between mothers and fathers, as well as between women and men (Table 2).^25,28^ Egberts et al found a higher prevalence at all time points (≤1 month, 3 months, and 12 months) for mothers than fathers, while Hawkins et al. found a higher prevalence for men than women at a single time point (≤2 months). It is worth noting that research focused on other conditions has consistently indicated that women generally assume greater caregiving responsibilities for their families and experience higher levels of stress, burden, and distress compared to men.^38,39^ These gender differences in caregiving roles and associated stress may contribute to the observed disparities in PTSS prevalence between mothers and fathers within the context of burn injuries.

Our review yielded studies exclusively in high-income countries. Mental health services can be limited in low- and middle-income countries for a variety of reasons, such as cultural beliefs and limited financial resources.^40^ Underreporting of mental issues could explain why data on PTSS and PTSD prevalence in parents of children who suffered a burn was notably absent for low- and middle-income countries in our review.

While conducting our study, we found that a previous review from 2013 examined the empirical literature on the psychological consequences of pediatric burns and discussed parental anxiety, traumatic stress, and depression in both the short and long term.^3^ However, our review differs in that it specifically focused on providing new and unique insights into PTSS and PTSD within 12 months of the burn injury, which has not been previously addressed. Additionally, a recently published (2021) meta-analysis by Wilcoxon et al. found a 17.5% prevalence for PTSD in parents following a burn injury to their children, with assessments conducted at various time points ranging from 1 month to approximately 8 years.^41^ Our review goes beyond these findings by providing more detailed and specific information on the effects within the 12 months after the burn injury. Furthermore, our review includes the assessment of PTSS in addition to PTSD, adding further depth to the analysis.

### Limits

Our review revealed that there is significant confusion around the terminology of PTSS and PTSD. In our study, we employed the PTSS (post-traumatic stress symptoms) definition, which included the main symptoms associated with PTSD. It encompassed characteristic symptoms such as reexperiencing traumatic events, avoiding reminders of the trauma and experiencing emotional numbness without being limited to the psychiatric diagnosis of PTSD.^18^ However, PTSS may also refer to post-traumatic stress syndrome, which has been defined previously as symptoms that are consistent with PTSD, but which manifest within a timeframe of less than 30 days following a traumatic incident.^42^ Only one of our included studies defined PTSS as post-traumatic stress syndrome,^28^ while another employed the terms PTSD, PTSS, and PTSD *symptoms* interchangeably when referring to a singular disorder.^16^ Alternatively, some studies used the same questionnaire but interpreted the results as PTSS or PTSD (eg Impact of Events Scale PTSS^17,18,24,25,29^; and PTSD^15^). Finally, some included manuscripts focused on evaluating the prevalence of PTSD *symptoms*.^15,33^ These articles were retained because the symptoms were used as a means to assess the diagnosis of PTSD. However, it is possible that other relevant articles may have been overlooked, as this specific terminology was not initially included in our research strategy. Moreover, certain articles referred to related psychiatric disorders and discussed acute stress disorder (ASD) or traumatic stress symptoms and could have been valuable in assessing parental distress in the context of burn injuries but were excluded (eg,^43^). Particularly, our search strategy yielded one excluded article reporting a prevalence of ASD of 2.2% in mothers and 0.7% in fathers within an average of 19 days following the burn injury to their child.^35^

Another limitation to consider is that our selection process was not restricted to studies focused solely on measuring the prevalence of PTSS or PTSD. As a result, our review may have included studies with broader primary objectives. As such, not all identified articles were informative and therefore included in the quantitative synthesis but were presented descriptively. Moreover, we did not restrict the articles we included to those where the prevalence was measured using questionnaires with validation evidence. By including studies without validation evidence for their questionnaires, there could be a risk of incorporating manuscripts with less reliable data, which may affect the overall robustness and accuracy of our review’s findings. Nonetheless, most of the included articles in our review assessed the validity and reliability of their chosen questionnaire. Many of our articles used the Impact of Event Scale (IES) or a version of this questionnaire, which has validation evidence as a screening tool for PTSD.^44,45^ We also included one article identified as having a high risk of bias for completeness, even though this article had an issue with exact measurement timing.^32^ Its inclusion did not, however, affect the interval of prevalence we reported. Finally, we cannot exclude the possibility of publication bias, which could affect our findings.

## CONCLUSION

In our study, we specifically focused on investigating the prevalence of PTSS and PTSD among parents within 12 months of their child’s burn injury. We found that the prevalence of PTSS ranged from 6% to 49%, while the prevalence of PTSD ranged from 4.4% to 22%. PTSS prevalence seemed to decrease over time. Considering the implications of our findings, it is crucial to recognize the importance of providing psychological support not only for burn patients but also for their parents, at least in the year following the traumatic event. This support can help address and alleviate the emotional and mental distress experienced by parents during this critical period. In turn, this can positively impact the child’s own recovery. Our review also draws attention to the significant confusion surrounding post-traumatic stress terminology within the scientific literature, emphasizing the need for clarification and standardization in this area of research.

## SUPPLEMENTARY MATERIAL

Supplementary material is available at *Journal of Burn Care & Research* online.

irae033_suppl_Supplementary_Appendix

## Notes

*Author Contributions:* Naomi Hope Chouinard (Data curation [Lead]; Formal analysis [Lead]; Investigation [Lead]; Methodology [Lead]; Project administration [Supporting]; Resources [Lead]; Software [Lead]; Validation [Lead]; Visualization [Lead]; Writing—original draft [Lead]; Writing—review & editing [Lead]), Davaine Joel Ndongo Sonfack (Data curation [Supporting]; Formal analysis [Supporting]; Software [Supporting]; Validation [Supporting]; Writing—review & editing [Supporting]), Sue-Ling Chang (Conceptualization [Supporting]; Formal analysis [Supporting]; Validation [Supporting]; Writing—review & editing [Supporting]), Frédéric Bergeron (Data curation [Supporting]; Software [Supporting]; Validation [Supporting]; Writing—review & editing [Supporting]), Chanel Beaudoin Cloutier (Conceptualization [Lead]; Project administration [Supporting]; Writing—review & editing [Lead]), Jason Guertin (Conceptualization [Lead]; Data curation [Lead]; Formal analysis [Lead]; Funding acquisition [Lead]; Investigation [Lead]; Methodology [Lead]; Project administration [Lead]; Resources [Lead]; Software [Lead]; Supervision [Lead]; Validation [Lead]; Visualization [Lead]; Writing—original draft [Lead]; Writing—review & editing [Lead])

*Funding:* This work was supported by the Quebec Cell, Tissue and Gene Therapy Network—ThéCell (a thematic network supported by the Fonds de recherche du Québec-Santé). NHC is recipient of a bursary from la Fondation des pompiers du Québec pour les grands brûlés and from the Fondation du CHU de Québec. J.R.G. is the recipient of a Chercheur-boursier Junior 2 career award from the Fonds de recherche du Québec-Santé.
